# Autofermentation of alkaline cyanobacterial biomass to enable biorefinery approach

**DOI:** 10.1186/s13068-023-02311-5

**Published:** 2023-04-08

**Authors:** Cigdem Demirkaya, Agasteswar Vadlamani, Taina Tervahauta, Marc Strous, Hector De la Hoz Siegler

**Affiliations:** 1 Department of Chemical and Petroleum Engineering, University of Calgary, Calgary, AB Canada; 2 Department of Geoscience, University of Calgary, Calgary, AB Canada

**Keywords:** Alkaliphiles, Anaerobic digestion, Fermentation, Cyanobacteria, Hydrogen

## Abstract

**Background:**

Carbon capture using alkaliphilic cyanobacteria can be an energy-efficient and environmentally friendly process for producing bioenergy and bioproducts. The inefficiency of current harvesting and downstream processes, however, hinders large-scale feasibility. The high alkalinity of the biomass also introduces extra challenges, such as potential corrosion, inhibitory effects, or contamination of the final products. Thus, it is critical to identify low cost and energy-efficient downstream processes.

**Results:**

Autofermentation was investigated as an energy-efficient and low-cost biomass pre-treatment method to reduce pH to levels suitable for downstream processes, enabling the conversion of cyanobacterial biomass into hydrogen and organic acids using cyanobacteria’s own fermentative pathways. Temperature, initial biomass concentration, and oxygen presence were found to affect yield and distribution of organic acids. Autofermentation of alkaline cyanobacterial biomass was found to be a viable approach to produce hydrogen and organic acids simultaneously, while enabling the successful conversion of biomass to biogas. Between 5.8 and 60% of the initial carbon was converted into organic acids, 8.7–25% was obtained as soluble protein, and 16–72% stayed in the biomass. Interestingly, we found that extensive dewatering is not needed to effectively process the alkaline cyanobacterial biomass. Using natural settling as the only harvesting and dewatering method resulted in a slurry with relatively low biomass concentration. Nevertheless, autofermentation of this slurry led to the maximum total organic acid yield (60% C mol/C mol biomass) and hydrogen yield (326.1 µmol/g AFDM).

**Conclusion:**

Autofermentation is a simple, but highly effective pretreatment that can play a significant role within a cyanobacterial-based biorefinery platform by enabling the conversion of alkaline cyanobacterial biomass into organic acids, hydrogen, and methane via anaerobic digestion without the addition of energy or chemicals.

**Supplementary Information:**

The online version contains supplementary material available at 10.1186/s13068-023-02311-5.

## Introduction

Cyanobacteria are promising biomass feedstocks to produce bioenergy and bioproducts, as they are fast-growing organisms with doubling times as low as 1.5 h [1]. A fast-growing cyanobacterial culture, however, can quickly deplete the dissolved inorganic carbon in the medium, leading to growth limitation. Carbon limitation can be addressed by operating at high pH and alkalinity, as under these conditions the CO_2_ mass transfer rate between air and the liquid medium is significantly enhanced, while the buffering capacity of the culture medium increases [2, 3]. Thus, more inorganic carbon is available to support photosynthesis and biomass growth, resulting in improved productivity [4, 5]. Fresh water has an alkalinity between 100 and 5000 µEq L^−1^ and a pH ranging from 6 to 9. Seawater has an alkalinity of about 2300 µEq L^−1^ and a pH of 8.2 [6]. A wide range of alkalinity has been used in prior studies to enhance biomass productivity [5, 7, 8], with alkalinity levels above 10,000 µEq L^−1^ (= 0.01 Eq L^−1^) considered as alkaline conditions. Protection against common competing organisms and predators have additionally been reported at alkalinities higher than 0.1 Eq L^−1^ [3]. In this report, we are concerned with the processing of cyanobacterial biomass produced at this higher end of the alkalinity gradient.

Alkalinity has a complex effect on the performance of downstream processes. For instance, high alkalinity can improve biomass degradability and biopolymers solubility, facilitating certain biomass conversion and product recovery operations [9–11]. On the contrary, the alkaline biomass slurry may be corrosive or may contain compounds that could have inhibitory effects or be considered as contaminants in the final products [12]. As downstream processes have a significant effect on the economic viability and environmental footprint of the produced biofuels or bioproducts, innovations in downstream processing must aim at minimizing energy inputs and avoiding the addition of chemicals.

The harvested biomass can be processed into several bioenergy products, such as biodiesel through the transesterification of neutral lipids, bioethanol through fermentation, biogas through anaerobic digestion, or bio-crude through thermochemical conversion [13]. Among these potential downstream processes, anaerobic digestion (AD) is attractive due to its low energy requirements and its ability to handle wet biomass, which eliminates the need for drying [14]. AD can also play a more general role within a biorefinery platform, as it can be used as a final step to convert any residual biomass into bioenergy (i.e., methane) at low cost and with high energy efficiency.

AD is a complex process involving four different stages: hydrolysis and acidogenesis, followed by acetogenesis and methanogenesis. The microorganisms more active in each stage differ in terms of their nutrient and pH requirements, physiology, growth, and tolerance to environmental stresses [15]. Although high pH and high alkalinity are beneficial for biomass cultivation, they are undesirable in all stages of AD due to their inhibitory effect [16]. Jiunn-Jyi et al. [17] investigated the effect of pH on the AD of activated sludge by changing the initial pH from 5.0 to 10.0. A significant decrease in methane production was reported when pH was above 8.3. Nolla-Ardevol et al. [18] proposed using microbial communities obtained from haloalkaline sediments to enable AD under high pH and high alkalinity. They reported that digestion of *Spirulina* under highly alkaline conditions (pH 10, 2.0 M Na^+^) into methane-rich (96%) biogas was possible. However, only 7% of the initial biomass was converted into methane due to the inhibitory effect of NH_3_-N and the accumulation of volatile fatty acids.

To enable the successful conversion of alkaline biomass slurries, a pretreatment method can be implemented to decrease the pH. However, direct neutralization is undesirable as it increases chemical usage and processing costs [19]. Dark anaerobic fermentation may be a suitable pretreatment, as it can result in the formation of acidic products and a reduction of pH. Towards this aim, it is relevant the work of Dahiya et al. [20] who reported acetic acid, butyric acid, and propionic acid production from food waste by dark fermentation under alkaline conditions (pH 10–11) and a subsequent reduction of pH.

Ananyev et al. [21] and Hasunuma et al. [22] have shown that *Spirulina platensis* and *Synechocystis* sp. can convert their own carbohydrates to a wide range of end products (*e.g.,* ethanol, formate, acetate, H_2_, and CO_2_). If this autofermentative metabolic capability is widely distributed among the cyanobacteria, then it may be possible to use it as a pretreatment step to reduce the pH of the cyanobacterial biomass slurry, reducing or eliminating the use of additional energy, catalysts, external organisms (bacteria or yeast), and nutrients [22–24]. Autofermentation can also become a key processing step within a biorefinery concept, allowing the production and recovery of chemical precursors and other valuable products.

In this report, we explore the feasibility of introducing a dark anaerobic fermentation step to enable the acidification of the highly alkaline biomass slurry harvested from cyanobacterial cultures grown at high alkalinity, while simultaneously performing the first two steps of anaerobic digestion, hydrolysis and acidogenesis. Furthermore, we combine the autofermentation with different harvesting techniques to reduce harvesting cost and evaluate the effect of several process parameters, such as initial biomass concentration and temperature, on the formation of acidic products, lowering of pH, and hydrogen formation during the autofermentation step. We also assess biogas production after autofermentation for the first time.

## Materials and methods

### Biomass production

The cyanobacterial culture used here consisted of a consortium dominated by *Candidatus* “Phormidium alkaliphilum”, an alkaliphilic and halotolerant cyanobacterium [25, 26]. This culture was derived by enrichment of microbial mats collected from several soda lakes located in British Columbia, Canada, as described by Sharp et al. [27]. Biomass was grown in cyclic batch mode using 10 L glass bottles under a photon flux of 900 µmol m^−2^ s^−1^ using full spectrum LED lamps (Hyperikon, HyperT5-4C-50). At the end of each batch, lasting 7 days, 95% of the culture medium was removed and replenished with fresh medium. The alkaline medium was prepared to a final pH of 10 and 0.5 mol L^−1^total alkalinity. This medium contained Na_2_CO_3_ (22.36 g L^−1^), NaHCO_3_ (6.54 g L^−1^), NaNO_3_ (340 mg L^−1^), MgSO_4_·7H_2_O (120 mg L^−1^), CaCl_2_ (19 mg L^−1^), NaCl (250 mg L^−1^), K_2_HPO_4_ (216 mg L^−1^), KCl (122 mg L^−1^), FeCl_2_ (5 mg L^−1^), ZnCl_2_ (20 µg L^−1^), MnCl_2_·4H_2_O (250 µg L^−1^), H_3_BO_3_ (600 µg L^−1^), CoCl_2_·6H_2_O (15 µg L^−1^), CuCl_2_·2H_2_O (15 µg L^−1^), NiCl_2_·6H_2_O (10 µg L^−1^), Na_2_MoO_4_·2H_2_O 15 µg L^−1^, and KBr (10 µg L^−1^).

### Biomass harvesting

At the end of each batch, biomass was harvested by either natural settling or centrifugation. Harvesting by natural settling was done by stopping mixing and allowing the culture to settle for up to 6 h. Samples were taken in triplicates at 1, 2, 3, and 6 h to monitor biomass concentration, settling efficiency, and used media removal. Centrifugation was carried at varying relative centrifugal force, applied for 15 min, for further dewatering of the harvested biomass. Total solids and volatile solid concentrations were determined after each harvesting procedure. The biomass paste harvested by centrifugation will be referred in this paper as solid-state condition as there was no free water, but it was moist enough to allow the fermentation process to happen.

### Fermentation

Approximately 2.0 g of the harvested biomass, either as a paste or slurry, were placed in sterile serum bottles. Serum bottles of varying volumes (20 mL, 100 mL, and 200 mL) were used for the different concentration experiments and sealed with rubber septa. The headspace in each bottle was vacuumed to 50 mbar and filled with argon gas up to atmospheric pressure to create anoxic conditions. Hypoxic conditions were created by sealing with a rubber septum without gas exchange. Incubation was performed under dark conditions to start respiration and eventually fermentation. The bottles were incubated at 21 ± 0.5 °C for up to 16 days. Every two days, three bottles were removed from the incubation chamber and analyzed as described in Sects. "Analysis of fermentation products" (liquid and solid phases) and (gas phase).

### Analysis of fermentation products

The pH of the fermented biomass was measured using a micro-pH probe (InLab, Mettler Toledo). The fermented biomass was either a slurry or a paste. In the latter case, the whole paste was resuspended in 5 mL of deionized water to extract organic acids and other soluble organic materials. In both cases, centrifugation for 15 min at 3894×*g* was used to separate the solid fraction containing cells and cell debris from the liquid phase. The supernatant was recovered and stored at – 80 °C, while the solid pellet was freeze dried and stored at – 20 °C for elemental analysis.

#### Liquid-phase analysis

The liquid samples recovered from the fermentation stage were filtered through a 0.2 µm PES sterile filter (Basix) and were diluted 5 to 15 times with deionized water (Type I, 18.2 MOh·cm) to get the concentrations of organic acids in the calibration range of 0.34 mM to 50 mM. Samples were analyzed by HPLC (Dionex ICS 3000, Thermo Fisher) using an Aminex HPX-87H (Bio-Rad) organic acid column (9 μm; 7.8 × 300 mm) and a UV–Vis detector. The HPLC was operated using 5 mM H_2_SO_4_ as mobile phase at a flow rate of 0.5 mL/min at a temperature of 35 °C. Standard calibration curves were prepared using acetic acid (Alfa Aesar, 99.7 + %), propionic acid (Acros Organics, 99%), formic acid (Acros Organics, 99%), butyric acid (Acros Organics, 99%), lactic acid (Acros Organics, 90%), and succinic acid (Alfa Aesar, 99.7 + %) as analytical standards.

Soluble sugar was analyzed by the sulfuric acid-phenol method; briefly, 50 μL of the sample were mixed with 150 μL of 98% sulfuric acid followed immediately by the addition of 30 μL of 5% phenol in water. After incubating for 5 min at 90 °C in a static water bath, the samples were cooled to room temperature for 5 min and absorbance at 490 nm was recorder with a SpectraMax ID3 microplate reader [28].

For soluble protein content analysis, 600 µL of the sample were mixed with 950 µL of the Lowry Reagent D and 0.1 mL of diluted Folin-Ciocalteu’s phenol reagent, then incubated for 30 min at room temperature. Absorbance was measured at 600 nm and protein concentration was obtained using a standard curve prepared with bovine serum albumin (VWR Life Science, 30% solution) [29].

Chemical oxygen demand (COD) was determined using the procedure described by Blaird et al. [30] using CHEMetrics COD vials (K-7355) with a range of 0–150 ppm.

Ammonium concentrations were determined using the OPA (*o*-phthaldiadehyde, Sigma P-1378) colorimetric assay as described by Holmes et al. [31].

#### Solid-phase analysis

The total solids and volatile solid concentration at every sampling point were determined using the method outlined by Wychen and Laurens [32].

Protein content of the initial biomass was extracted and determined by the method described by Slocombe et al. [29].

Elemental composition of the harvested biomass was determined using a CHN analyzer (2400 Series II, Perkin Elmer), using acetanilide as calibration standard.

### Carbon recovery and hydrogen yield

The carbon recovery, *R*, was calculated based on:

1 $$R=\frac{{\sum }_{i=1}^{m}{C}_{c,i}\times V}{{C}_{c,\mathrm{biomass},t=0}},$$

where *C*_*c*_ is the carbon content of component *i* at time *t*, *V* is the sample volume, and *m* is the number of measured carbon-containing compounds.

The maximum theoretical hydrogen yield (*Y*_HB_, in mL H_2_/g biomass) was calculated as:

2 $${Y}_{\mathrm{HB}}={Y}_{\mathrm{HM}}\times {X}_{M}$$

where *Y*_HM_ is the stoichiometric hydrogen yield of monosaccharides (497.8 mL H_2_/g monosaccharides), and *X*_*M*_ is the total monosaccharides concentration in the biomass [33].

### Anaerobic digestion

Batch anaerobic digestion was performed in 200 mL serum bottles, with continuous agitation at 150 rpm in an incubator operating at 35 °C. The methanogenic inoculum used in these tests was obtained from an industrial-scale anaerobic digester treating activated sludge from a municipal wastewater treatment plant (Bonnybrook Wastewater Treatment Plant, Calgary, Canada) operating at mesophilic (35 °C) conditions. The inoculum and biomass were mixed at ratio of 2 on COD basis and the working volume was fixed at 120 mL. The bottles were sealed, and the headspace in each bottle was vacuumed to 50 mbar and filled with argon gas up to atmospheric pressure to create anoxic conditions. The pressure increase in the headspace during incubation was measured using an Omega DPG4000 series digital manometer.

### Gas-phase analysis

An air-tight syringe (Hamilton Company) was used to collect gas samples from the incubation vials' headspace. Samples were injected into a Varian GC to determine hydrogen and methane concentration. The instrument was equipped with a porous polymer column (10ʹ × 1/8″ OD SS, 80/100 Mesh), a Molecular Sieve column (10ʹ × 1/8″ OD SS, 80/100 Mesh), and a thermal conductivity detector. Argon (99.999%, Air Liquide) was used as the carrier gas, running at 30 mL/min with column oven temperature at 100 °C and the TCD operating at 120 °C.

## Results and discussion

### Feasibility of anaerobic digestion for processing alkaline cyanobacterial biomass

Cyanobacterial biomass cultivated at high pH and alkalinity was harvested via centrifugation. The harvested biomass paste, with a pH of 10.48 ± 0.02, was left to auto-ferment statically in the dark for 10 days at 21 °C. Next, activated sludge was added to the autofermented paste and the mixture was incubated anoxically for 40 days to stimulate anaerobic digestion and biogas production.

Harvested biomass paste was used directly as a control in the anaerobic digestion experiments. During digestion, the pressure in the gas head space was monitored to determine biogas production (Additional file 1: Fig. S1). No biogas production was observed in the incubations of untreated alkaline biomass. On the other hand, the dark fermentation pretreatment step decreased the biomass pH from 10.48 to 6.90, due to the accumulation of organic acids. In this case, the pressure in the head space during AD incubations increased to 3.84 ± 0.35 bar, and the headspace consisted of 61.84 ± 0.25% methane (314.15 mL/g AFDM).

Ammonium concentration increased significantly during AD of both autofermented and untreated biomass. It must be noted that the freshly harvested cyanobacterial biomass was determined to have a protein content of 60.9%. This high protein content reduces the C:N ratio of the biomass and leads to high ammonia production from amino acids degradation [34]. During anaerobic digestion, the ammonium concentration increased steadily from 17 to 90 mM and 126 mM in the untreated and autofermented biomass, respectively. Even though the ammonium concentration during digestion of autofermented biomass was higher than during digestion of untreated biomass, it did not appear to cause inhibition. At high pH, a large part of the ammonium is present as the free base, ammonia (NH_3_). Ammonia easily permeates cell membranes and can increase intracellular pH, causing inhibition [35].

### Optimization of dark fermentation conditions

Based on the previous results, dark autofermentation shows to be a feasible treatment for alkaline cyanobacterial biomass, enabling its successful anaerobic digestion by reducing the pH. During autofermentation, several organic acids accumulated while proteins and other cellular materials were released into the growth medium. To further explore autofermentation as a potential element of a biorefinery concept, experiments were performed to determine the effect of fermentation conditions on product distribution.

#### Effect of temperature

**Fig. 1:**
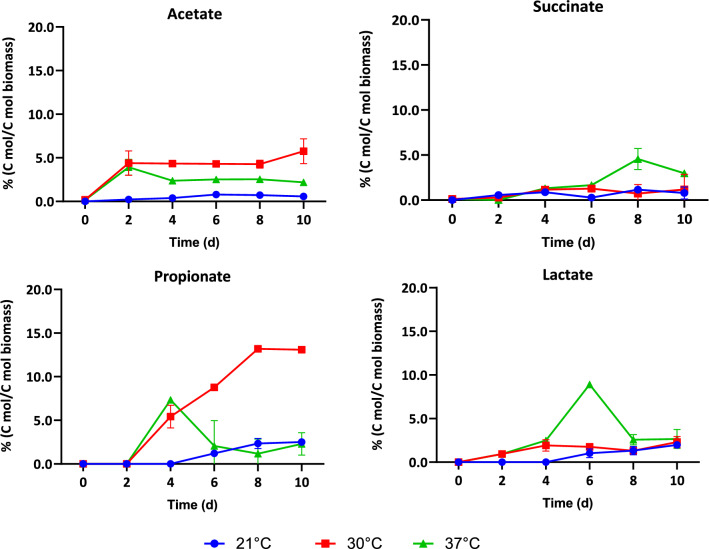
The effect of fermentation temperature on organic acid yield during anoxic dark fermentation. Initial pH in all cases was 10.36 ± 0.05. Values reported corresponding to the average of triplicate measurements ± 95% confidence interval

**Fig. 2:**
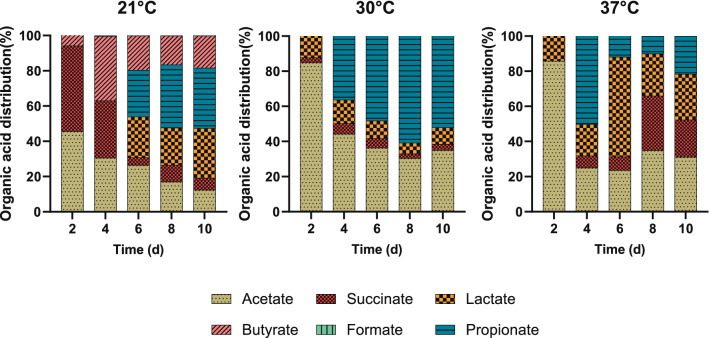
Organic acid product distribution at different fermentation temperatures. Initial pH in all cases was 10.36 ± 0.05

Static fermentation of the biomass paste harvested by centrifugation was conducted at different temperatures (21, 30, and 37 °C). Six organic acids: acetate, propionate, butyrate, formate, succinate, and lactate, were detected as fermentation products at 21 °C. Butyrate and formate were not detected at 30 and 37 °C. The organic acid yield and distribution obtained at different temperatures are shown in Figs. 1 and 2.

The yield of each organic acid increased significantly as the operating temperature increased from 21 to 30 °C (Fig. 1). For all temperatures, the highest total organic acid yield was obtained between days 6 and 8. The highest total organic acid yield was obtained at 30 °C on day 8. The yields at 21 °C were significantly different from those at higher temperatures for acetate (*P* = 0.009), butyrate (*P* = 0.02), and lactate (*P* = 0.0005). The highest succinate and lactate yields were obtained at 30 °C on day 8; however, the relative abundance of these two acids at 30 °C was lower than at 21 °C. Thus, organic acid recovery may be simpler at the lower fermentation temperature.

The soluble protein increased rapidly at 30 °C and 37 °C, reaching its maximum at day 2 and decreasing afterwards (Additional file 1: Fig. S3). This indicates that higher temperature resulted in an increase in the rate of both protein release and protein degradation. At 21 °C, there was a steady increase in the total soluble protein, reaching the highest concentration at day 6. Thus, low fermentation temperature may be beneficial as it will allow to maximize the recovery of protein.

The fractional recovery of carbon in fermentation products provides a measure of the efficiency of the bioconversion. A higher carbon recovery indicates that most of the carbon initially present in the biomass has been transformed into desired products. Carbon recovery at different temperatures after 8 days of fermentation is shown in Table 1.

**Table 1:** Carbon balance for the autofermentation of highly alkaline cyanobacterial biomass at different temperatures

	Carbon recovery (mg C/g initial AFDM)^a^		
	21 °C	30 °C	37 °C
Initial total carbon in biomass	500.95	507.81	508.27
Final total carbon in biomass	231.01	258.38	251.61
Total carbon in organic acids	35.62	98.99	55.22
Acetate carbon	3.67	21.74	12.95
Succinate carbon	4.54	3.64	23.17
Formate carbon	1.22	n.d	n.d
Butyrate carbon	7.83	n.d	n.d
Propionate carbon	11.76	66.95	5.99
Lactate carbon	6.60	6.66	13.12
Total sugar carbon	5.73	3.73	9.22
Total protein carbon	98.71	81.79	46.63

^a^ Carbon distribution results are reported after 8 days under dark anaerobic conditions

At 30 °C, the carbon conversion into soluble organic acids was maximized, reaching 19%. At this same temperature, 87% of the carbon was recovered in the form of organic molecules. The remainder evolved as CO_2_ and other non-measured products, including free amino acids. The lowest total carbon recovery was at 37 °C, where only 71.3% of the carbon was accounted for; while, at 21 °C most of the solubilized carbon was in the form of solubilized proteins (19.7%) and only about 7.1% of the carbon was present as organic acids. Depending on the relative value of the different fermentation products, fermentation temperature can be adjusted to maximize the recovery of the most valuable ones. Moreover, as organic acid production reaches its peak at day six, fermentation could be stopped at that time if the goal is to maximize organic acid recovery.

#### Effect of initial solids concentration

In Sect. "Feasibility of anaerobic digestion for processing alkaline cyanobacterial biomass", we established that static fermentation is a suitable pretreatment to enable the anaerobic digestion of the alkaline cyanobacterial biomass paste. Although this pretreatment offers several advantages compared to suspended cultures, including a reduction in water utilization and easiness for recovering high value by-products, it requires an energy intensive and expensive step for harvesting and dewatering, such as centrifugation or membrane filtration. These steps increase the solid fraction in the harvested biomass up to 27% at the expense of high energy use of up to 8 kWh m^−3^ [36, 37]. The higher energy requirement of centrifugation accounts for up to 40% of the total operating costs in some algal production systems [36, 38]. Consequently, it is desirable to replace centrifugation with a low energy harvesting technique.

Several low energy techniques such as flocculation, flotation, filtration, and sedimentation, or a combination of any of these, are used to harvest and concentrate microalgal biomass [39]. Biomass concentration can be increased from dilute solid concentrations (0.1–0.26%) to 3–10% solid concentration by settling, flocculation, or filtration, which have energy consumption in the range of 0.1–0.4 kWh m^−3^ [38].

Flocculation and sedimentation can be used as a primary method to decrease the cost of harvesting [37]. Some microbes can form large settleable flocs as a result of co-precipitation with ions at high pH, and cell–cell interactions capable of self-flocculation, forming large settleable colonies, and enabling simple and effective separation by gravity sedimentation.

Water recovery and solid concentration after natural settling and centrifugation are reported in Additional file 1: Figure S2. The biomass concentration (as ash-free dry mass, AFDM) in the culture medium at harvesting was 0.75 ± 0.09 g/L. Approximately, 97.6% of the water was recovered after settling, reaching a final biomass concentration of 30.96 ± 2.52 g/L. Biomass concentration could be further increased to 50 g/L by applying low-speed centrifugation at 48×*g* for 15 min. To reach a concentrated paste (219 g/L), biomass was centrifuged at 3894×*g* for 15 min.

Next, we evaluated the impact of these harvesting and dewatering methods on the outcomes of the autofermentation. Different harvesting and dewatering methods resulted in changes in the concentration of the harvested biomass, also modifying the total carbonate concentration and buffering capacity of the autofermented slurry. For a fixed biomass load (AFDM basis), the total fermentation volume differs for each harvesting method. Although the carbonate concentration is the same across all harvesting methods, the amount of medium relative to solids differs. Thus, harvesting methods that result in a more concentrated slurry or paste have lower ratio of medium relative to solids, and this results in a lower buffering capacity. In Table 2, the maximum total organic acid yield and final pH after 8 days of fermentation at 21 °C is reported for biomass harvested via primary settling (t_settling_ = 1 h), secondary settling (t_settling_ = 2 h), low-speed centrifugation (RCF = 48×*g*), and high-speed centrifugation (RCF = 3894×*g*).

**Table 2:** Effect of harvesting method on the performance of autofermentation of alkaline cyanobacterial biomass.^a^

	High-speed centrifugation	Low-speed centrifugation	Secondary Settling	Primary settling
Biomass concentration (g AFDM/L)	219.36 ± 3.94	47.05 ± 3.95	10.21 ± 0.36	3.04 ± 0.16
Volume (mL)	2.00	8.76	43.8	146
Initial pH	10.48 ± 0.02	10.48 ± 0.02	10.48 ± 0.02	10.48 ± 0.02
Final pH	6.87 ± 0.06	8.48 ± 0.06	9.80 ± 0.01	10.25 ± 0.01
Maximum organic acid yield (mmol/g AFDM)	0.91 ± 0.15	1.51 ± 0.22	2.43 ± 0.19	10.28 ± 0.80
Organic acid peak day	10	10	10	8

^a^ Results reported correspond to fermentation at 21 °C, under anoxic conditions

Even though the initial pH was the same for all cases, the centrifuged paste had less buffering capacity due to lower total amount of carbonates and resulted in a rapid decrease in pH, reaching neutral values (7.19 ± 0.07) within 2 days. At the lowest biomass concentration obtained by natural settling, the decrease in pH was much smaller. The small pH change was due to the higher total carbonates and corresponding higher buffering capacity. The final pH at the lowest biomass concentration was 10.31 ± 0.01, which may still be inhibitory for methanogenesis as suggested by the results shown in "Feasibility of anaerobic digestion for processing alkaline cyanobacterial biomass" section.

**Fig. 3:**
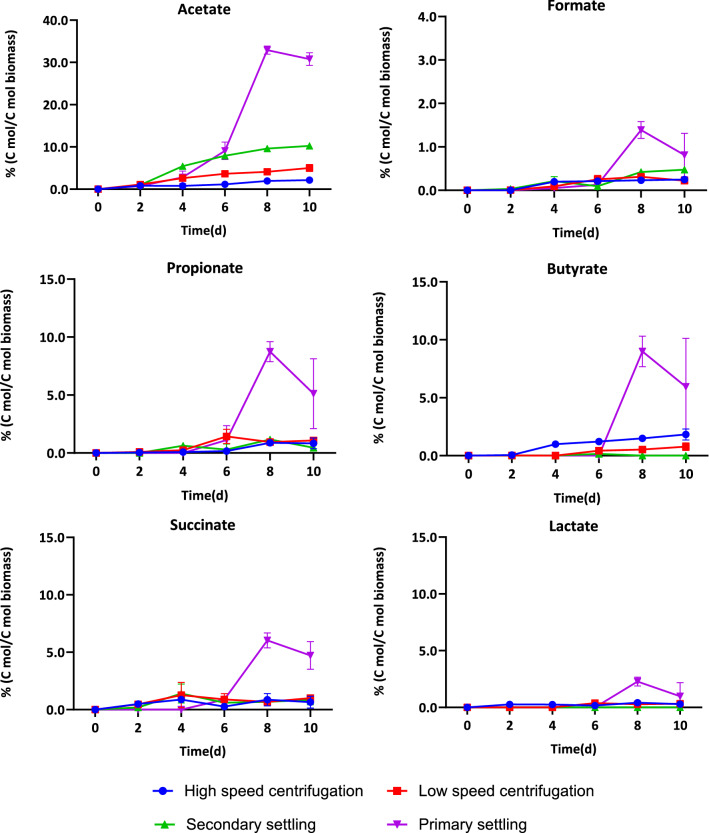
The effect of harvesting and dewatering method on organic acid yields (mmol-organic acids produced per g-initial biomass) during 10 days of anoxic dark fermentation. Initial pH in all cases was 10.48 ± 0.02. Values reported corresponding to the average of triplicate measurements ± 95% confidence interval

**Fig. 4:**
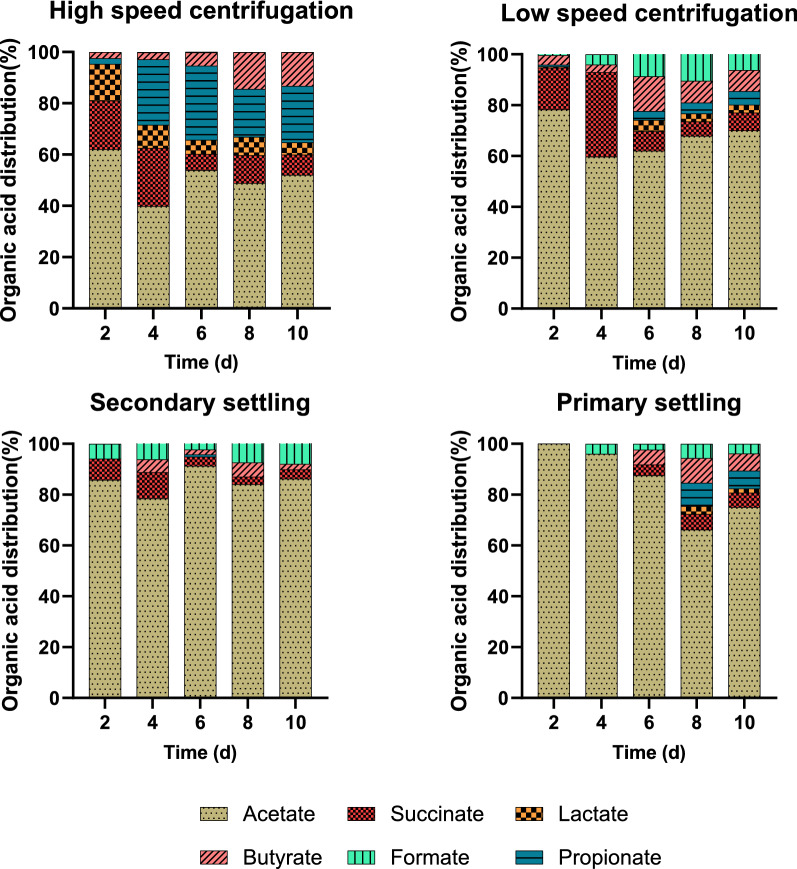
Organic acid product distribution at different concentration of highly alkaline and high pH media during 10 days of anoxic dark fermentation. Initial pH in all cases was 10.48 ± 0.02

The effect of initial biomass concentration on organic acid yields and organic acid composition profile is shown in Figs. 3 and 4, respectively.

At the lowest solids concentration (3.04 g/L), obtained with primary settling, the organic acid production was more than 14-fold higher than at other biomass concentrations. Acetate was the main product, with significant amounts of butyrate, propionate, and succinate co-produced. At the highest biomass concentration, there was a more balanced profile of produced organic acids. Acetate was still the most abundant, accounting for an average of 53% of the total acids.

During dark fermentation, the complex storage molecules of the cells are first hydrolyzed to monomers. The production and accumulation of organic acid is dependent on monomer availability. High pH is beneficial for the solubilization of complex molecules, enabling their conversion and increasing organic acid yields [40]. In the case of biomass harvested by primary settling the yield of soluble proteins and sugar was higher than for other harvesting methods, indicating that high pH promoted faster biomass hydrolysis (Additional file 1: Figure S4).

The highest acetate yield was obtained at the lowest initial biomass concentration (Fig. 3). Acetate production has been previously shown to be higher under alkaline conditions than at neutral or low pH [41]. As acetate is an important precursor for methanogenesis that can contribute 60–70% of methane generation, an increased conversion of biomass into acetate may be beneficial for increasing methane production.

Propionate and butyrate were the second and the third most abundant organic acids at higher biomass concentrations. Propionate and butyrate serve as intermediate fermentation products and, with additional incubation time, may be converted into acetate for further methane production during anaerobic digestion.

The highest carbon recovery was observed at the lowest initial biomass concentration, with 60.4% of the initial carbon being recovered as organic acids (Additional file 1: Table S1), and about 26.5% of the carbon present as solubilized proteins. Thus, lower initial biomass concentration favored the conversion of alkaline cyanobacterial biomass slurries during dark fermentation, resulting in higher organic acid yields obtained with lower dewatering energy needs.

#### Effect of the presence of oxygen

**Fig. 5:**
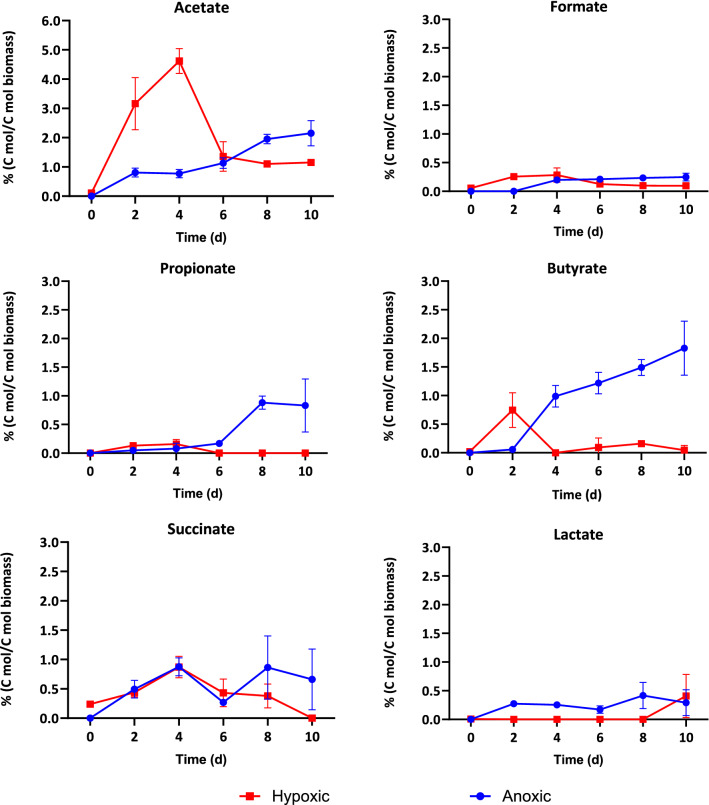
Organic acid yields (mmol-organic acids produced per g-initial biomass) during 10 days of anoxic and hypoxic dark fermentation. Initial pH in all cases was 10.35 ± 0.01. Values reported corresponding to the average of triplicate measurements ± 95% confidence interval

**Fig. 6:**
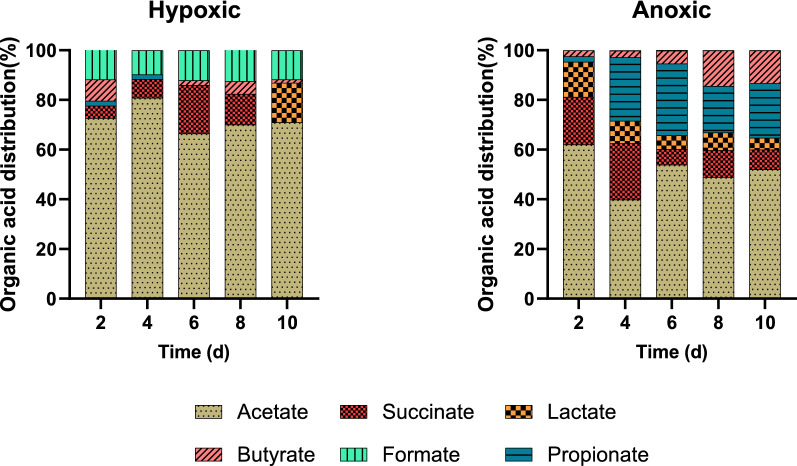
Organic acid percentage and product distribution under hypoxic and anoxic highly alkaline and high pH media during 10 days of dark fermentation. Initial pH in all cases was 10.35 ± 0.01

The cyanobacteria used in this study have significant metabolic flexibility allowing them to adapt to fluctuating redox conditions [25]. However, little is known regarding how the cell metabolism reacts to changing oxygen levels and whether oxygen affects organic acids production and product distribution. To elucidate this effect, we studied the organic acid yield and distribution during dark fermentation of cyanobacterial biomass under conditions of anoxia (0% O_2_ in gas headspace) and hypoxia (2% O_2_ in gas headspace) as shown in Figs. 5 and 6.

The presence of oxygen significantly increased acetate and formate yields, while yields for butyrate, propionate, and lactate were higher under anoxic conditions. On the other hand, formate was not detected under anoxic incubations. There was a more balanced distribution of organic acids under strictly anoxic conditions.

Similar organic acid carbon yield was obtained under hypoxic and anoxic conditions with 33 and 30.6 mg carbon per gram biomass respectively (Additional file 1: Table S2). However, complete anoxia resulted in a longer lag phase. The organic acid yield reached its peak at 4–6 days under hypoxic conditions, whereas this peak was delayed to day 8 under anoxic conditions. The longer lag phase observed under strictly anoxic conditions is likely the result of a longer adaptation phase needed when transitioning from oxic illuminated conditions in the photobioreactor to strict anoxia in the fermenter.

### CO_2_ and hydrogen production

Carbon dioxide (CO_2_) is a common by-product during fermentation. The high alkalinity of the fermentation medium, however, results in low or no release of CO_2_ as most of it will remain dissolved as bicarbonate or carbonate. Of the initial carbon present in the biomass, only 2.2% and 0.12% was released as CO_2_ during autofermentation at 21 °C of the biomass obtained by high-speed centrifugation and low-speed centrifugation, respectively. No CO_2_ release was observed at the lower concentrations obtained by natural settling. Increasing fermentation temperature translated into an increased CO_2_ production at 30 °C and 37 °C of the biomass obtained by high-speed centrifugation with 16.4% and 21.5% of the initial carbon being released as CO_2_, respectively. Changes in fermentation oxygen content did not measurably affect the release of CO_2_.

Interestingly, analysis of the produced gases by GC revealed the production of hydrogen. NADH is produced during dark fermentation as part of the cell metabolic activities. NADH needs to be recycled as NAD^+^ to continue supporting ATP production via glycolysis. Hydrogenases and nitrogenases catalyze the reversible reduction of protons to H_2_ coupled to NAD^+^ regeneration in some cyanobacteria genera [21]*.* The cyanobacterium used in this study have the genes encoding for uptake hydrogenase (*hypABCD*), bidirectional hydrogenase (*hoxEUS, hndC*), and nitrogenase (NifK, NifD, NifH,) [25]. Thus, it is possible that the production of hydrogen during autofermentation is part of the metabolic response of *Candidatus* “Phormidium alkaliphilum”.

The maximum stoichiometric hydrogen yield of biomass was calculated as 119.5 mL of H_2_ per gram of biomass, based on the total sugar and protein concentration determined for the biomass and assuming that no other products, other than CO_2_ and H_2_, were produced. Maximum hydrogen production occurred at 21 °C and at the lowest biomass concentration (3.04 g/L). Under these conditions, a H_2_ yield of 326.14 µmol/g AFDM was found, corresponding to 6.1% of the maximum stoichiometric yield.

The increased hydrogen production at the lowest initial biomass concentration may be caused by the higher overall pH associated with autofermentation at the lowest biomass concentration. As previously indicated, harvesting via primary settling results in a slurry containing a lower ratio of biomass to total carbonates, which translates to a higher buffering capacity and a lower drop in pH during fermentation (see Table 2). Ananyev et al. [21] showed that, in the cyanobacterium *Arthrospira maxima,* hydrogen production is a process operating close to thermodynamic equilibrium under biologically relevant conditions. By removing the produced hydrogen from the culture medium, they were able to increase the net rate of hydrogen formation. As the solubility of hydrogen in aqueous medium decreases with increasing concentration of carbonate or bicarbonate ions [42], the higher carbonate amount present at the lowest biomass concentration may explain the increased hydrogen production. Additionally, as no CO_2_ was released at the lowest biomass concentration, this resulted in the accumulation of pure H_2_ in the headspace.

### Autofermentation role within a biorefinery platform

Autofermentation effectively converts highly alkaline cyanobacterial biomass into organic acids and hydrogen. To make the overall process more economically feasible, it is important to valorize most of the fermentation products. Produced hydrogen can be separated from the headspace, while the fermentation broth can be separated into an organic acid rich fraction and a solid fraction. Furthermore, valorization of the organic acids to high value-added products (e.g., polyhydroxyalkanoates) is possible, while the remaining solid fraction can be processed into biogas using AD. Separation of the liquid phase, including solubilized proteins, also eliminates rapid ammonium accumulation in the anaerobic digestion.

Table 3 presents a summary of the organic acid yield and titers reached during the autofermentation of microalgal and cyanobacterial biomass. For ease of comparison with previous studies, organic acid yields shown in Table 3 are reported in g/g DM.

**Table 3:** Organic acid yield and final product concentration obtained via autofermentation of microalgal and cyanobacterial biomass

Microorganism	Organic acid yield (g/g DM)	Organic acid concentration (g/L)	Fermentation Conditions			Harvesting	Initial biomass concentration (g DM/L)	Incubation time (days)	Reference
			Temperature	pH	Added Chemicals				
*Arthrospira maxima*	0.55	0.825	30 °C	9.80	210 mM Na^+^	Filtration	1.5	2	[43]
*Arthrospira platensis*	0.011	0.22	35 °C	–	NaH_2_PO_4_/Na_2_HPO_4_ buffer solution	Filtration	50	5	[44]
*Chlorella sorokiniana*	0.30	7.5	29–31 °C	10.40	Carbonate/bicarbonate buffer	Centrifugation	25	2	[45]
*Nannochloropsis* sp.	0.061	–	38 °C	–	–	Centrifugation	11–29 wt%	1	[46]
*Candidatus* “P. alkaliphilum”^a^	0.619	2.16	21 °C	10.48	–	Natural settling	3.04	8	This study
*Candidatus* “P. alkaliphilum”^b^	0.064	15.37	21 °C	10.48	–	Centrifugation	219.36	8	This study

^a^ Autofermentation at the lowest initial biomass concentration (3.04 g AFDM/L)

^b^ Autofermentation at the highest initial biomass concentration (219.36 g AFDM/L)

In general, the fermentation of microalgal and cyanobacterial biomass have been shown to result in low titers, especially when compared to the fermentation of carbohydrate-rich biomasses [47]. Microalgal and cyanobacterial biomass typically have a low carbohydrate to protein content and given that carbohydrates are the primary substrates for fermentation [48], the relatively low total organic acid titers are an expected result.

Compared to previous studies (see Table 3), harvesting by natural settling resulted in the highest organic acid yield at 0.616 g/g DM. This present study also corresponds to the lowest fermentation temperature, demonstrating a successful pathway for converting alkaline biomass to organic acid yield with minimal energy inputs.

The highest total organic acid concentration of 15.37 g/L, on the other hand, was obtained under solid-state fermentation conditions, albeit at lower temperatures than the previous studies. The energy needs and cost of recovery and purification decrease as the product concentration increases. As there is a clear trade-off between higher yields and titers, proper bioprocess design will require to strike a balance between the initial biomass concentration (i.e., how much energy is spent in dewatering) and the final organic acid concentration (i.e., how much energy is spent in product recovery).

## Conclusions

Autofermentation is a simple, but highly effective pretreatment to enable the conversion of alkaline cyanobacterial biomass into methane via anaerobic digestion without the addition of energy or chemicals. In addition, it results in the production of high value added bioproducts and hydrogen. In this study, we reported autofermentation of natural mixed cyanobacterial biomass. Here, we obtained the highest organic acid yield at the lowest biomass concentration, demonstrating that extensive, energy intensive, dewatering is not needed. Although the presence of oxygen affects the organic acid yield and distribution, strict anoxia is not needed to promote the autofermentation of alkaline cyanobacterial biomass. The successful conversion of cyanobacterial biomass into multiple products using a simple and energy-efficient process was demonstrated, however, further studies are required to optimize overall processing conditions and economics.

## Supplementary Information

**Additional file 1: Figure S1.** Change in pressure and accumulated methane concentration obtained from inoculation of untreated and treated highly alkaline and high pH microalgal biomass with activated sewage sludge inoculum during 30 and 40 days of incubation respectively. Methane concentration was detected with gas chromatography at different time points of the incubation. Experiments were performed in triplicates and statistical analysis were done with two-way Anova test with a significance level of 0.05. **Figure S2.** Change in biomass concentration by natural settling for 6 h (a) and centrifugation at different speed for 15 min (b) and water recovery efficiency. **Figure S3.** The effect of fermentation temperature on soluble protein and sugar (mg per g-initial biomass) during 10 days of anoxic dark fermentation. Initial pH in all cases was 10.36 ± 0.05. Values reported correspond to the average of triplicate measurements with 95% confidence interval. **Figure S4.** The effect of harvesting and dewatering method on soluble protein and sugar (mg per g-initial biomass) during 10 days of anoxic dark fermentation. Initial pH in all cases was 10.48 ± 0.02. Values reported correspond to the average of triplicate measurements with 95% confidence interval. **Table S1**. Carbon balance for the autofermentation of highly alkaline cyanobacterial biomass at different initial biomass concentrations. Carbon distribution results are reported after 8 days under dark anaerobic conditions. **Table S2.** Carbon balance for the autofermentation of highly alkaline cyanobacterial biomass under hypoxic and anoxic conditions at 21 °C. Carbon distribution results under hypoxic and anoxic are reported after 4 and 8 days respectively.

## Data Availability

All data generated or analyzed during this study are included in this published article and its Additional files.
