# Supplementary material for: Autofermentation of alkaline cyanobacterial biomass to enable biorefinery approach
Source: Biotechnol Biofuels Bioprod. 2023 Apr 8;16:62. doi: 10.1186/s13068-023-02311-5 (PMC10082510; doi:10.1186/s13068-023-02311-5)
Supplement: Supplementary file 1 — Additional file 1: Figure S1. Change in pressure and accumulated methane concentration obtained from inoculation of untreated and treated highly alkaline and high pH microalgal biomass with activated sewage sludge inoculum during 30 and 40 days of incubation respectively. Methane concentration was detected with gas chromatography at different time points of the incubation. Experiments were performed in triplicates and statistical analysis were done with two-way Anova test with a significance level of 0.05. Figure S2. Change in biomass concentration by natural settling for 6 h (a) and centrifugation at different speed for 15 min (b) and water recovery efficiency. Figure S3. The effect of fermentation temperature on soluble protein and sugar (mg per g-initial biomass) during 10 days of anoxic dark fermentation. Initial pH in all cases was 10.36 ± 0.05. Values reported correspond to the average of triplicate measurements with 95% confidence interval. Figure S4. The effect of harvesting and dewatering method on soluble protein and sugar (mg per g-initial biomass) during 10 days of anoxic dark fermentation. Initial pH in all cases was 10.48 ± 0.02. Values reported correspond to the average of triplicate measurements with 95% confidence interval. Table S1. Carbon balance for the autofermentation of highly alkaline cyanobacterial biomass at different initial biomass concentrations. Carbon distribution results are reported after 8 days under dark anaerobic conditions. Table S2. Carbon balance for the autofermentation of highly alkaline cyanobacterial biomass under hypoxic and anoxic conditions at 21 °C. Carbon distribution results under hypoxic and anoxic are reported after 4 and 8 days respectively. [file 13068_2023_2311_MOESM1_ESM.docx]

**Supplementary Material To:**

**Autofermentation of alkaline cyanobacterial biomass to enable biorefinery approach**

Cigdem Demirkaya^1^, Agasteswar Vadlamani^2^, Taina Tervahauta^1^, Marc Strous^2^, Hector De la Hoz Siegler1^[[1]](#footnote-2)^

^1^Department of Chemical and Petroleum Engineering, University of Calgary, Calgary, AB, Canada.

^2^Department of Geoscience, University of Calgary, Calgary, AB, Canada.

Corresponding author: [h.siegler@ucalgary.ca](mailto:h.siegler@ucalgary.ca) (H. De la Hoz Siegler).


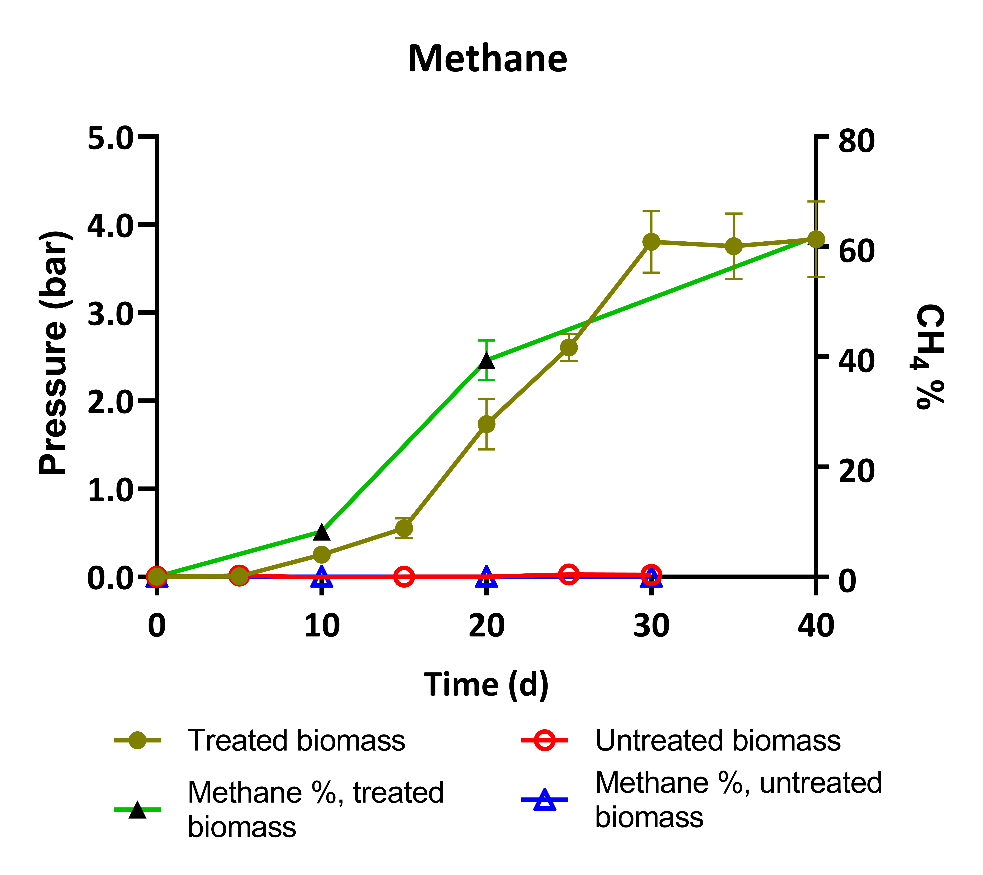
Fig.S1 Change in pressure and accumulated methane concentration obtained from inoculation of untreated and treated highly alkaline and high pH microalgal biomass with activated sewage sludge inoculum during 30 and 40 days of incubation respectively. Methane concentration was detected with gas chromatography at different time points of the incubation. Experiments were performed in triplicates and statistical analysis were done with two-way Anova test with a significance level of 0.05.


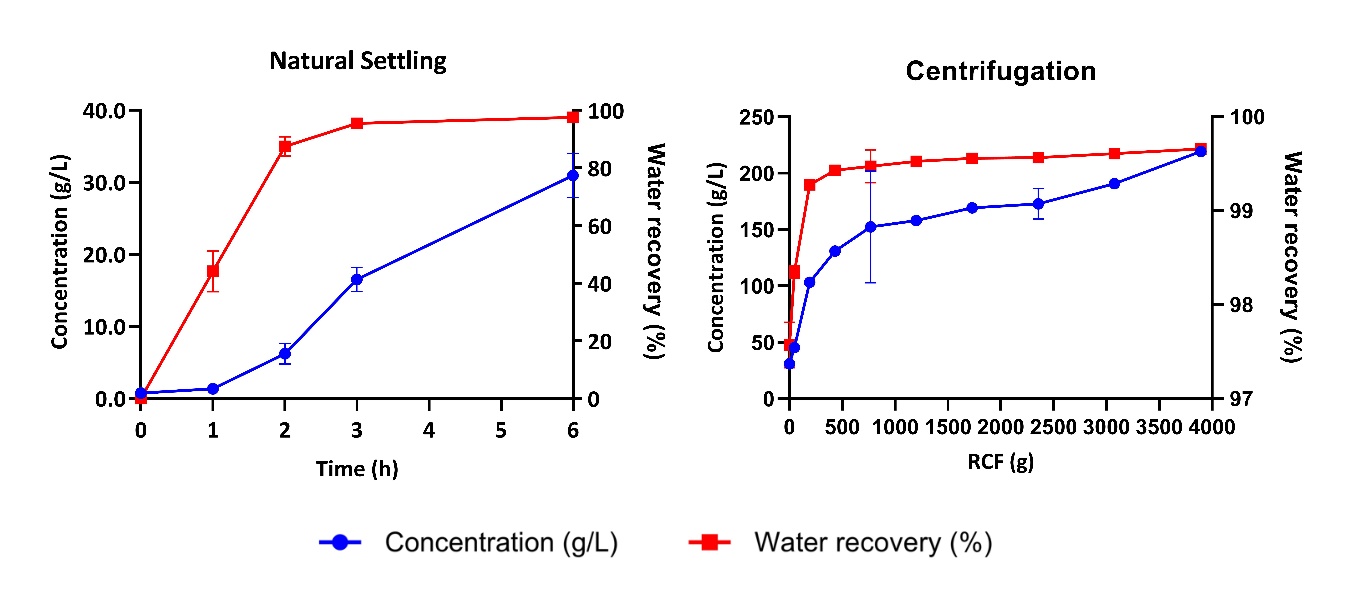
Fig. S2. Change in biomass concentration by natural settling for 6 hours (a) and centrifugation at different speed for 15 min (b) and water recovery efficiency.


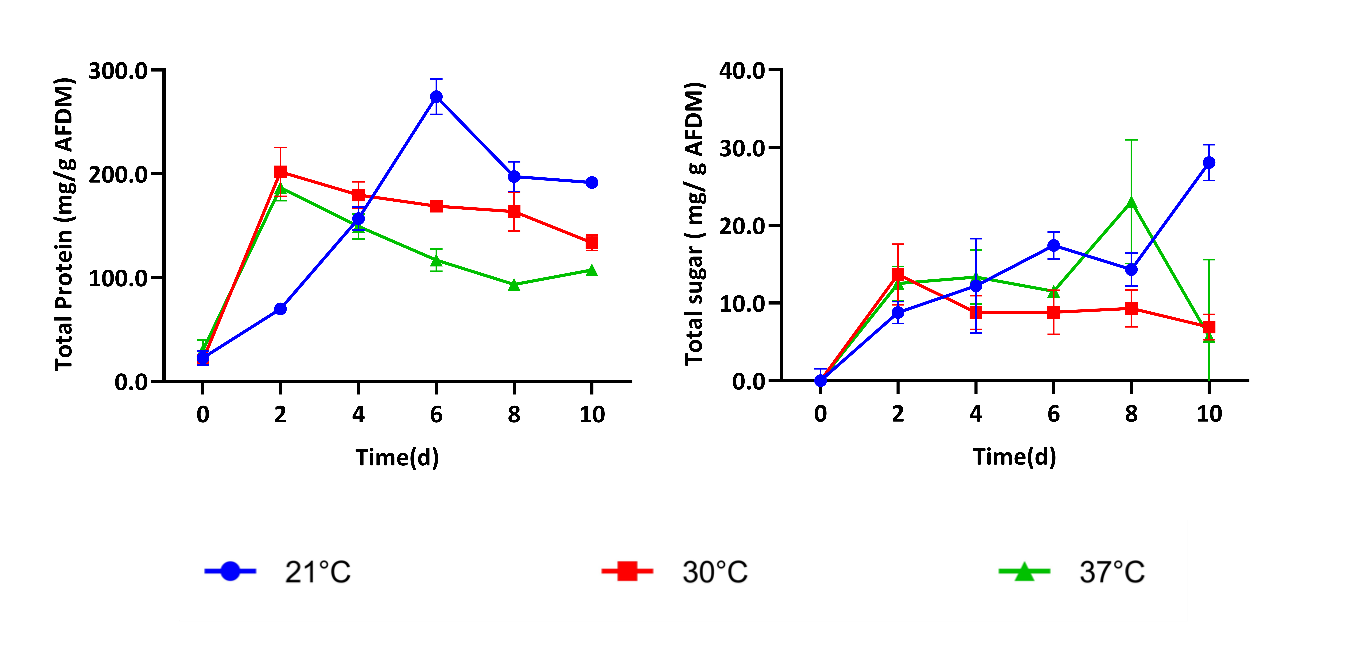
Fig. S3. The effect of fermentation temperature on soluble protein and sugar (mg per g-initial biomass) during 10 days of anoxic dark fermentation. Initial pH in all cases was 10.36 ± 0.05. Values reported correspond to the average of triplicate measurements with 95% confidence interval.


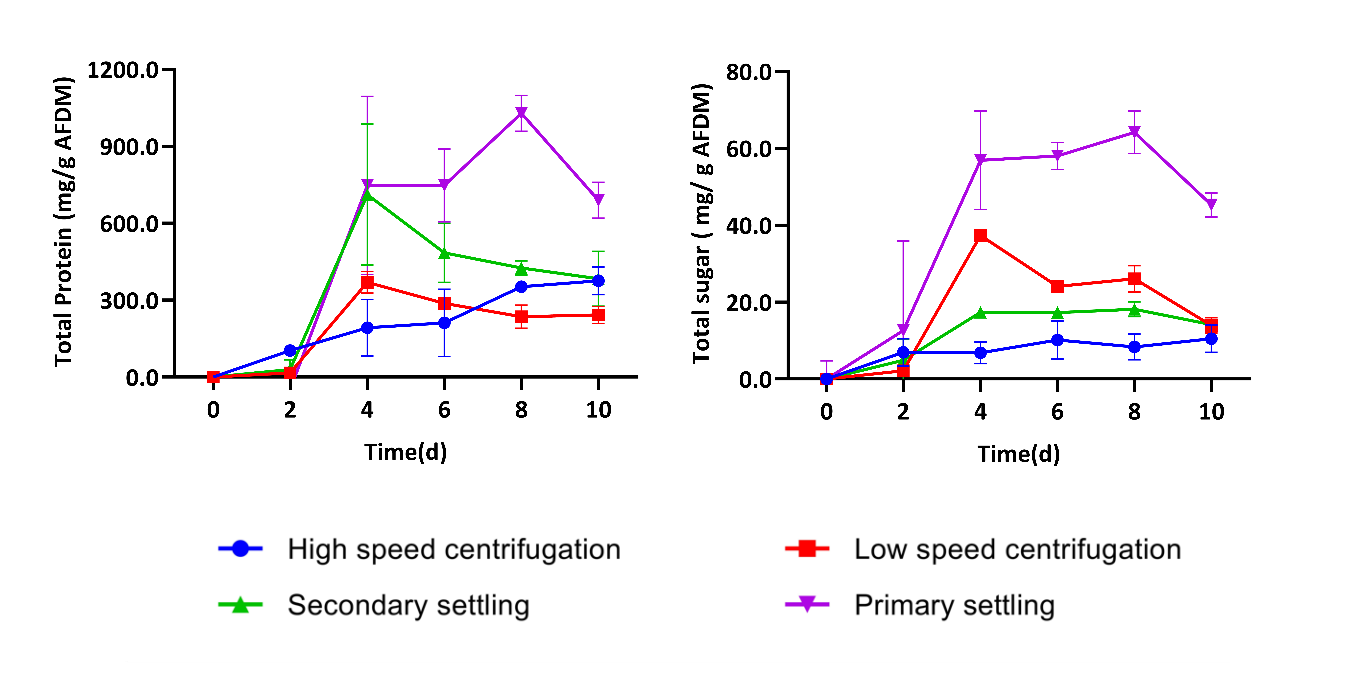


Fig. S4. The effect of harvesting and dewatering method on soluble protein and sugar (mg per g-initial biomass) during 10 days of anoxic dark fermentation. Initial pH in all cases was 10.48 ± 0.02. Values reported correspond to the average of triplicate measurements with 95% confidence interval.

Table S1. Carbon balance for the autofermentation of highly alkaline cyanobacterial biomass at different initial biomass concentrations. Carbon distribution results are reported after 8 days under dark anaerobic conditions.

|  | **Recovered carbon** (mg/g initial AFDM) | | | |
| --- | --- | --- | --- | --- |
|  | High Speed Centrifugation | Low Speed Centrifugation | Secondary Settling | Primary Settling |
| ***Initial total organic carbon in biomass*** | 524.55 | 524.55 | 495.06 | 495.06 |
| ***Total organic carbon in biomass at day 8*** | 310.10 | 395.60 | 142.94 | 80.43 |
| ***Total organic acid carbon*** | 30.61 | 35.98 | 58.83 | 298.81 |
| ***Acetate carbon*** | 10.24 | 21.54 | 47.60 | 162.95 |
| ***Succinate carbon*** | 4.53 | 3.59 | 3.38 | 29.85 |
| ***Formate carbon*** | 1.22 | 1.65 | 2.08 | 6.86 |
| ***Butyrate carbon*** | 7.82 | 2.77 | n.d | 44.53 |
| ***Propionate carbon*** | 4.63 | 4.95 | 5.77 | 43.29 |
| ***Lactate carbon*** | 2.18 | 1.48 | n.d | 11.33 |
| ***Total sugar carbon*** | 4.17 | 13.05 | 9.08 | 32.08 |
| ***Total protein carbon*** | 183.09 | 122.79 | 260.91 | 221.57 |

Table S2. Carbon balance for the autofermentation of highly alkaline cyanobacterial biomass under hypoxic and anoxic conditions at 21°C. Carbon distribution results under hypoxic and anoxic are reported after 4 and 8 days respectively.

|  | **Recovered carbon** (mg/g initial AFDM) | |  |
| --- | --- | --- | --- |
| Component | Hypoxic | Anoxic | |
| ***Initial total organic carbon in biomass*** | 539.72 | 524.55 | |
| ***Total organic carbon in biomass*** | 388.70 | 310.10 | |
| ***Total organic acid carbon*** | 33.12 | 30.61 | |
| ***Acetate carbon*** | 25.78 | 10.24 | |
| ***Succinate carbon*** | 4.87 | 4.53 | |
| ***Formate carbon*** | 1.58 | 1.22 | |
| ***Butyrate carbon*** | n.d. | 7.82 | |
| ***Propionate carbon*** | 0.88 | 4.63 | |
| ***Lactate carbon*** | n.d. | 2.18 | |
| ***Total sugar carbon*** | 1.72 | 13.04 | |
| ***Total protein carbon*** | 46.90 | 183.09 | |

1. Correspondence: [h.siegler@ucalgary.ca](mailto:h.siegler@ucalgary.ca) (H. De la Hoz Siegler). [↑](#footnote-ref-2)
